# Low Temperature Nanoindentation: Development and Applications

**DOI:** 10.3390/mi11040407

**Published:** 2020-04-13

**Authors:** Shunbo Wang, Hongwei Zhao

**Affiliations:** 1 School of Mechanical and Aerospace Engineering, Jilin University, Changchun 130025, China; wang.shun.bo@163.com; 2 Key Laboratory of CNC Equipment Reliability, Ministry of Education, Changchun 130025, China

**Keywords:** low temperature, nanoindentation, hardness test, apparatus, mechanical properties

## Abstract

Nanoindentation technique at low temperatures have developed from initial micro-hardness driving method at a single temperature to modern depth-sensing indentation (DSI) method with variable temperatures over the last three decades. The technique and implementation of representative cooling systems adopted on the indentation apparatuses are discussed in detail here, with particular emphasis on pros and cons of combination with indentation technique. To obtain accurate nanoindentation curves and calculated results of material properties, several influence factors have been carefully considered and eliminated, including thermal drift and temperature induced influence on indenter and specimen. Finally, we further show some applications on typical materials and discuss the perspectives related to low temperature nanoindentation technique.

## 1. Introduction

Nanoindentation technique has become one of the most popular mechanical property testing methods in recent years [1,2,3,4,5]. Compared with uniaxial tension and compression tests, the specimen requirements of nanoindentation were more simplified: a flat, no shape required surface. The mechanical properties, including but not limited to hardness and elastic modulus, can be obtained automatically via commercial system using Hertz contact theory [6] and Oliver-Pharr analysis method [7]. Moreover, selectable micro-region and huge testing amount on the single specimen provides significant advantages of nanoindentation over other macro-mechanical tests. Mechanical properties of each component of the specimen can be studied separately in details, as well as the interaction between them [8,9]. The negligible testing area of nanoindentation is also in favor of high throughput test and testing on small-scale materials, such as films and particles [10,11].

With attaching low temperature environment on the conventional nanoindentation system, materials can be investigated at service temperatures and conditions relevant to industrial applications via nanoindentation method. Scientifically, low temperature nanoindentation technique is attractive to academic researchers for its combination low temperature environment to convenient nanoindentation method. The researchable aspects are not only limited to variation of specific mechanical parameters with temperature decreasing, while high hydrostatic pressure, shear stress, and local strain can be easily realized via nanoindentation method [12,13,14]. Interesting phenomena caused by such reasons are also strongly associated with the variation of temperature. Combining with subsequent testing analysis procedures on residual impressions, such as focused ion beam (FIB), scanning electron microscope (SEM), and transmission electron microscope (TEM) methods, the complex evolvement caused by nanoindentation at low temperatures can be observed intuitively [15,16,17,18].

Low temperature nanoindentation has developed in nearly the past three decades. However, the investigations on such field are not as much as high temperature nanoindentation [19,20,21,22,23] and relevant commercial system was relatively rare. Compared with various and flexible ways of application of in a high temperature environment, including resistance and laser heating, basically all the cooling sources in low temperature nanoindentation system are cryogenic liquids, such as liquid nitrogen (LN_2_) and liquid helium (LHe) [24,25,26,27]. Academic designers are always dedicated to solving the difficulties in applying a low temperature environment on a nanoindentation system and retaining the high sensitivity and accuracy of nanoindentation technique. 

In this article, the methods and testing theories of indentation at ambient and low temperature, including micro-hardness testing and nanoindentation testing, are introduced. Several stages of nanoindentation systems capable of testing at low temperatures are discussed in detail. The major design elements featured in the systems are described in relation to their low temperature functionality. Influence factors leading to measurement error, including contact thermal drift and variation of testing system itself under a low temperature environment, are also presented. Current application status of low temperature indentation technique is introduced in testing materials respects: metallic materials, semiconductor materials, and ceramic materials. A summary of the development on low temperature nanoindentation techniques and perspectives of the technique are concluded.

## 2. Methods of Indentation

Ever since people found the mechanical properties of material can be investigated artificially, different kinds of testing methods have been developed through destroying the original structure of the specimen. An impression can be obtained on the surface of one material permanently by another sharp object through a certain contacting load. The size of the impression was logically related with the “hardness” of the impressed material, which became the origin of the modern indentation technique. In this section, we will introduce working principles of two different indentation techniques, micro-hardness and nanoindentation, which are widely applied in academic research, and analysis of the two techniques, respectively, will be discussed.

### 2.1. Micro-Hardness Testing

**Figure 1:**
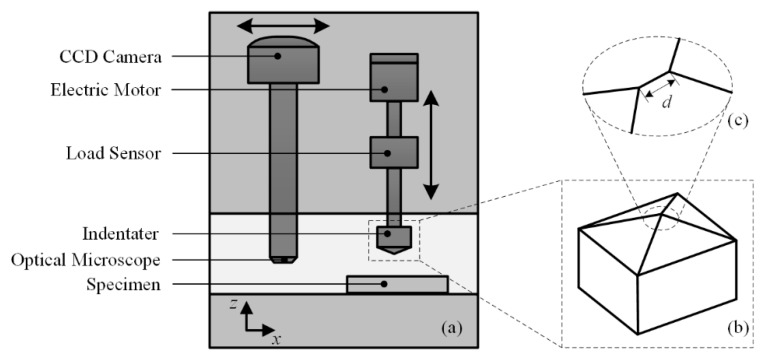
Schematic diagram of a micro-hardness system. CCD—charge coupled device.

A representative system of micro-hardness test is shown in Figure 1a, containing electric motor, load sensor, indenter, and optical microscope. During an indentation process, the electric motor drives the indenter moving down along z axis until the reading of the load sensor reaches target value. After a few seconds containing a target load (within 10 s), the indenter rises up from the surface of the specimen. Then, the optical microscope moves along the x axis to replace the position of indenter and enables researchers to observe the impression caused by indenter through a charge coupled device (CCD) camera. 

Micro-hardness technique is highly inseparable from macro-hardness technique, where the only difference is the testing load range applied by indenter. In general, the tests with indentation load between 9.8 × 10^−3^ N and 1.961 N are referred to as micro-hardness process, while macro-hardness process is usually conducted over 49.03 N [28]. It can be easily found that the value of the indentation load are all integer multiple of gravitational acceleration (~9.807 m/s^2^). This is due to a historical precedent that people used standard weights (like 1 kg and 10 kg) to apply the indentation load at the early stage of hardness technique. Even though the standard weights have been widely replaced by load sensors at present, people still adopt the multiple or semi-multiple value of gravitational acceleration to conduct micro-hardness tests. 

**Figure 2:**
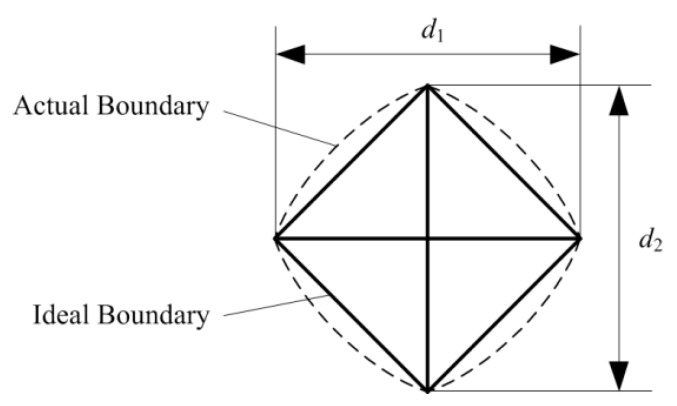
Schematic diagram of residual impression.

The testing procedure of micro-hardness testing is succinct and provides ideas for the subsequent nanoindentation testing technique. The indenters used in micro-hardness testing are generally Vickers indenter and Knoop indenter, while the former is more common. The tip of the Vickers indenter is a pyramid with four geometrically symmetric edges, where the angle between the opposite faces is 160°. Figure 2 gives the schematic diagram of residual impression conducted by Vickers indenter. Under ideal conditions, the shape of the impression is a square, and the lengths of diagonal (d1 and d2) can be calculated through the relative position of the vertexes observed by optical microscope. 

The Vickers hardness number, in terms of N and m, is calculated as follows [28]:

(1) $$HV=\frac{P}{A}=\frac{2P\sin\left(\alpha /2\right)}{d^{2}}$$

(2) $$HV=1.8544\times \frac{P}{d^{2}}$$

where:

*P*—indentation force, N;
*A*—area of the residual impression, m^2^;
*d*—mean diagonal length of the impression, m; and
*α*—face angle of the Vickers indenter, 68°.

During the above calculation process, people assume that there is no recovery occurred when the indenter is removed after loading cycle. That is, it is assumed that the indentation retains the shape of the indenter after the force is removed, which is fundamentally different from the nanoindentation introduced in the next section. On the other hand, the size of the residual impression can be extremely small when the indentation load is tiny. In general, it is suitable to adopt an optical microscope measuring the length of diagonals over 20 μm, while the observation of diagonal length under 10 μm needs the assist of SEM. However, the greatest strength of micro-hardness technique is the convenient and intuitive testing procedure, while the observation under SEM complicated the hardness acquisition process through micro-hardness. Thus, researchers devoted to find a more efficient method to obtain the hardness of material under tiny indentation load, and the nanoindentation was developed in succession.

### 2.2. Depth-Sensing Nanoindentation Testing

#### 2.2.1. Nanoindentation System 

Nanoindentation testing technique was essentially developed on the basis of micro-hardness technique, with attaching an additional displacement detecting sensor of indenter and converting the load sensor with smaller range, as called depth-sensing indentation (DSI) technique [29]. The nanoindentation systems can be mainly divided into two different types, based on driving principles: electrostatic force and converse piezoelectric effect [30,31,32]. 

**Figure 3:**
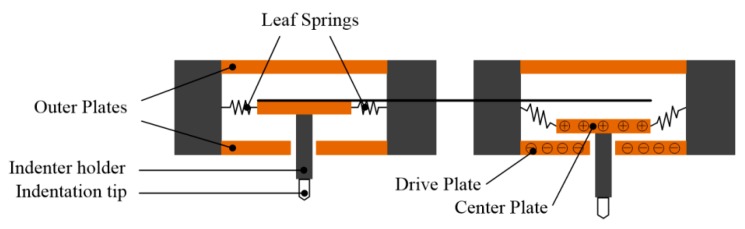
Schematic diagram of electrostatic driving method.

The electrostatic driving method is based on the interaction between charged electrode plates, as shown in Figure 3, in which exertion of the load and monitor of the displacement can be realized through one single component: three-plate capacitive transducer. The resolution and minimum indentation load can be extremely tiny, adopting the electrostatic force driving method as 100 μN in theory, which becomes the basis of basically all the current investigations with maximum indentation load under 1 mN. 

**Figure 4:**
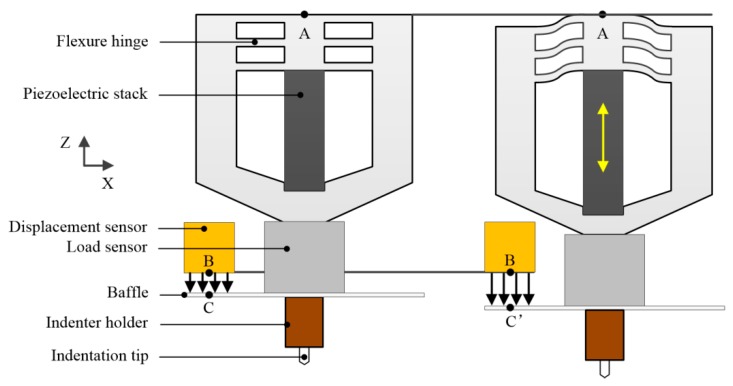
Schematic diagram of piezoelectric driving method [33].

By contrast, the method driven by piezoelectric ceramic is more suitable for researchers tending to build experimental platform by themselves, as shown in Figure 4. The recording data processes of load and displacement are separated through load sensor and capacitive displacement sensor, respectively, leading to the reduction of the complexity of the assembly and the precise of the involved units in contrast with electrostatic force method. Meanwhile, the maximum indentation load can also be raised largely to 1N (even 10 N), which has an overlapping range with micro-hardness testing. 

Besides the different driving principles, an integrated nanoindentation system usually contains several essential components:

- Macroscopic motion unit: commonly actuated by electric motors to realize the millimeter scale motion, including the rapid approaching of the indenter to the surface of specimen along z axis, and the position change of the specimen in xy plane. Actually, this unit is quite similar to the motion unit in micro-hardness testing system.
- Loading unit: actuated by electrostatic capacity, magnetic coils, or piezoelectric stack to realize the nanometer scale motion of indenter along the z axis, including the pre-touch process (right before the touch moment between indenter and specimen, while the reading of the load sensor is zero) and indentation process.
- Acquisition unit: mainly referring to load and displacement sensor, which are the most essential parameters during nanoindentation experiment and subsequent data analysis process. The data of load and displacement should ideally be pertained to indenter and embodies the superiority combined with micro-hardness technique.
- Observation unit: a set of optical microscope is usually provided in commercial nanoindentation system. Different from the dimension measurement of residual impression in micro-hardness testing, the microscope in nanoindentation technique is mainly used for determine indentation region of the specimen, as the size of the residual impression conducted by nanoindentation is so tiny.
- Control system: receiving and processing the signal from load and displacement sensors on computer. Meanwhile, the loading unit is also controlled to adjust the loading rate in real time during the whole indentation procedure. The experimental results, including hardness and elastic modulus, are analyzed and calculated by control system after indentation testing.
- Indenter: realizing the impressing process during indentation process.

The indenter mainly involves Vickers indenter, Berkovich indenter (three pyramids), cube corner indenter, spheroconical indenter, and flat-ended indenter, corresponding to the different research purpose. However, the most widely used Vickers indenter in micro-hardness is currently substituted by Berkovich type in nanoindentation tests. The ineluctable line of junction occurred between opposite faces (Figure 1c) during the manufacturing process in Vickers indenter seriously affected its application in tiny load tests.

#### 2.2.2. Nanoindentation Theories

**Figure 5:**
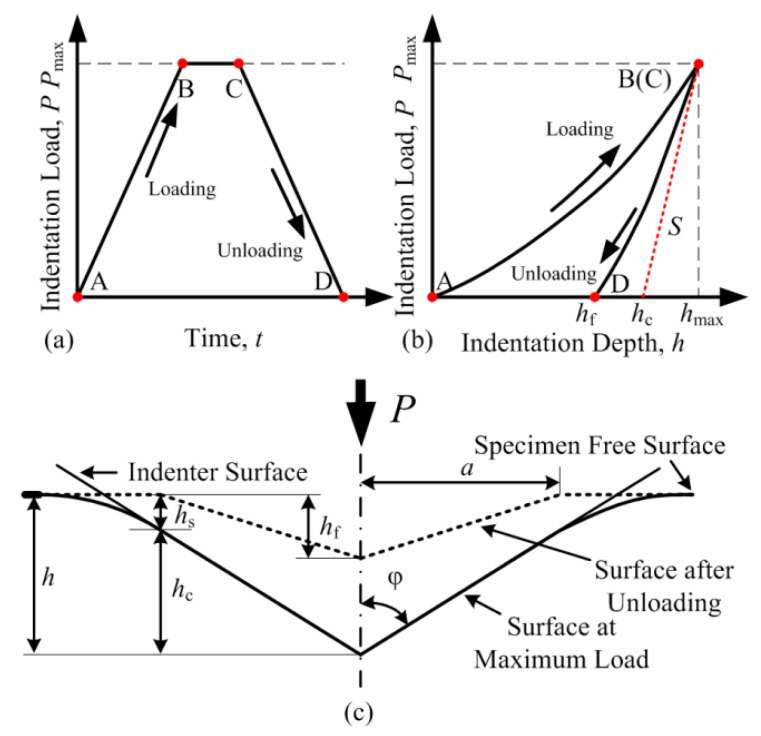
Schematic diagram of nanoindentation. (**a**) Typical load-time (*P*-*t*) curve. (**b**) Typical load-displacement (*P-h*) curve. (**c**) Physical meaning of parameters in calculation process.

The initial purpose of development of nanoindentation technique can be regarded as utilizing the data obtained by load and displacement sensors to calculate the mechanical properties, instead of direct measurement of the size of residual impressions in micro-hardness testing. Benefitting from the real time acquisition of load sensor and proportional-integral-derivative (PID) control of load unit through computer, nanoindentation testing can realize a load-control indentation process, as shown in Figure 5a. The corresponding indentation curve (*P-h* curve) is shown in Figure 5b. 

In the past several decades, theories of nanoindentation have been developed to calculate mechanical properties basing on *P-h* curve. Oliver-Pharr model is the most representative and basis of most other analysis methods [7,29]. It is assumed that the loading part of indentation is an elastic-plastic contact process, while the unloading part is purely elastic. Thus, not only the hardness, but the elastic modulus of specimen can also be obtained through unloading curve. The relationship formulated between indentation load *P* and displacement *h* during the unloading process (CD section in Figure 5b) is considered as a power law relation:

(3) $$P=\alpha {\left(h-h_{f}\right)}^{m}$$

where *h*_f_ is the depth of residual depth after indentation, and *α* and *m* are power law fitting constants related to specific unloading curve. The stiffness *S*, which is related to overall elastic recovery of during the unloading process, can be calculated by the slope at the vertex of the unloading curve:

(4) $$S={\frac{dP}{dh}|}_{h=h_{max}}$$

For calculation of hardness *H*, the true indentation depth *h*_c_ (the vertical distance between the tip of indenter and the point where indenter and surface of specimen separated at maximum indentation depth) is calculated as:

(5) $$h_{c}=h_{\max}-h_{s}=h_{\max}-\varepsilon \frac{P_{\max}}{S}$$

where *h*_s_ is the elastic deformation around indentation region and will recover to flat surface after unloading, and *ε* is a constant only relation with the shape of indenter. Then, the hardness *H* of specimen can be calculated as:

(6) $$H=\frac{P_{\max}}{A}$$

where *A* is the true projected contact area between indenter and surface, and it can be calculated from *h*_c_ and geometry of indenter. The relationship between *A* and *h*_c_ of ideal indenter is:

(7) $$A=\xi h_{c}^{2}$$

where *ξ* is a constant only relation with the shape of indenter (Berkovich, *ξ* = 24.56). 

For calculation of elastic modulus *E*, the reduced modulus *E*_r_ (combined modulus of the indenter and specimen) can be determined as:

(8) $$E_{r}=\frac{\sqrt{\pi }}{2\beta }\frac{S}{\sqrt{A}}$$

where *β* is a constant related with the shape of indenter. The elastic modulus of specimen can be given as:

(9) $$\frac{1}{E_{r}}=\frac{1-\nu_{i}^{2}}{E_{i}}+\frac{1-\nu^{2}}{E}$$

where *ν* is the Poisson’s ratio of specimen, and *ν*_i_ and *E*_i_ are the Poisson’s ratio and elastic modulus of indenter, respectively. 

Through Oliver-Pharr method, researchers can effectively obtain mechanical properties of material without directly observing the residual impression under microscope. However, the calculation of the hardness and elastic modulus is indirect and involves calculated parameters. For instance, the obtaining process of hardness essentially relies on the estimation of residual contact area, which follows the calculation method of micro-hardness testing. The determination of *h*_s_ and *A* can be intensely influenced by specific phenomenon during the indentation process, e. g., pile-up and creep. Thus, several correction methods have been proposed to improve Oliver-Pharr method to adapt different application scenarios [34,35,36]. The same situation also occurs in low temperature nanoindentation testing, which will be discussed in following, Section 4. 

## 3. Technical Development of Low Temperature Nanoindentation System

A low temperature environment is relative to the ambient temperature (room temperature (RT) 293 K), generally referring to the temperature range between RT and absolute zero (0 K). It has several call methods for the division of temperature value: cold, low, cryogenic (referring in particular to temperature below 120 K), and ultra-low temperature, while researchers choose the specific name according to their investigation temperature range [37,38,39]. In this article, “low temperature” is employed to describe temperatures below RT universally in order to simplify the writing. Generally, the core of the realization of a low temperature environment is cryogenic technology, such as liquid vaporization refrigeration, thermoelectric refrigeration, and magnetic refrigeration [40,41,42]. Low temperature state can be applied on the subject and/or atmosphere which researchers hope to refrigerate. In the field of material testing, a low temperature environment is most frequently exerted on the uniaxial tensile tests, which is conducive to understand strength, elasticity, and plasticity macroscopically [43,44,45]. Correspondingly, indentation technology attached with a low temperature environment provides the possibility to investigate micro and nano mechanical properties of materials. The current developed low temperature indentation apparatuses can be generally divided into two types according to the principle of refrigeration: system immersed in cryogenic liquid and contact-type low temperature testing.

### 3.1. System Immersed in Cryogenic Liquid

**Figure 6:**
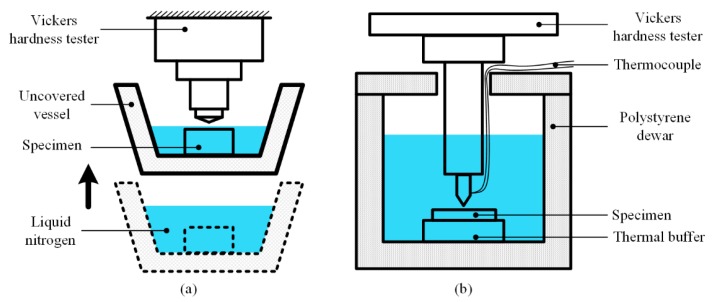
Schematic diagram of indentation test immersed in liquid nitrogen (LN_2_). (**a**) Original setup. (**b**) Improved setup.

The very earliest indentation testing combined with a low temperature environment can be traced back to the 1960s. Marsh et al. attempted to utilize liquid nitrogen (LN_2_) to cool down and obtain a low temperature specimen [46], as shown in Figure 6a. An uncovered vessel was used to contain LN_2_ and specimen, while the specimen was fully immersed into the liquid. As the boiling point of the LN_2_ is 76.59 K (~77 K) under atmospheric pressure, the temperature of the specimen immersed in LN_2_ is generally considered as constant 77 K. To conduct the indentation process, a normal Vickers hardness tester was firstly employed. Limited by the space of the Vickers indenter, researchers had to wait for the LN_2_ naturally evaporating until leaving only a thin layer over the specimen surface, and then raise the vessel and specimen rapidly to the Vickers indenter to conduct indentation. 

However, a considerable flaw of such a method was found that a great deal of heat flowed from the RT diamond indenter to the specimen, inducing that the indentation area was warmed up during contact. To eliminate the temperature diffusion problem, researchers expanded the distance between specimen and liquid surface, while the Vickers indenter was immersed into the LN_2_ before the indentation process. The connection between the indenter and the hardness testing machine was Tufnol rod, which effectively prevented heat flowing to indenter from upper upsets. 

Kurkjian et al. improved the LN_2_ immersed method by substituting polystyrene dewar (with a lid) and phenol fiber rod for common uncovered vessel and Tufnol rod, respectively [24], as shown in Figure 6b. Meanwhile, a chromel-alumel thermocouple was attached to the indenter with a small area to monitor testing temperature. To a certain extent, these improvements increased the accuracy of indentation tests. However, the additive thermocouple only played a role of monitoring, but not controlling the temperature. The utilization of LN_2_ limited the testing on a specific temperature point, and research has showed that the sudden low temperature applied on the specimen usually caused cracks. More importantly, the temperature control of LN_2_ is relied on the boiling point, leading to a constant boiling phenomenon. The produced nitrogen bubbles resulted in turbulence inside the dewar and partial bubbles directly stroked the indenter, which affected the measurement of load during the indentation process. 

**Figure 7:**
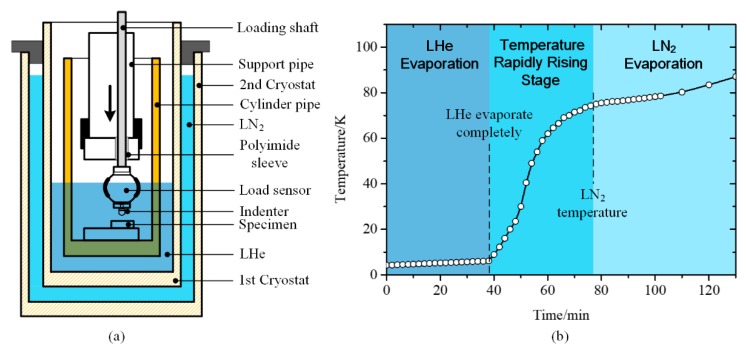
(**a**) Schematic diagram of 4.2 K indentation device. (**b**) Realization of temperature variation of specimen [47]. LHe = liquid helium.

An apparatus with lower indentation temperature was developed in Niigata University in 1996, which realized hardness testing at 4.2 K [47]. Figure 7a only shows schematic diagram of the cryogenic assembly and indentation region of the apparatus, as the overall height is ~2 m. The unexpressed parts are related mechanical devices, which drive the indenter vertically approaching and applying the load, as well as the specimen rotating in the horizontal plane to change the indentation area, respectively. The specimen and indenter were immersed in liquid helium (LHe, with a boiling point of 4.2 K) in a cryostat. To reduce the consumption of LHe, an extra cryostat filled with LN_2_ was placed outside the 1st cryostat as a cold shield. The specimen was fixed on the bottom of a cylinder pipe, while the indenter and load sensor were placed at the end of a loading shaft, which is supported by a polyimide sleeve inserted in a support pipe. Though friction was inevitable between the shaft and sleeve, the load sensor was separate beneath the friction area and unaffected from the friction. Additionally, the test temperature was actually controlled by liquid type, resulting in three available temperatures (4.2 K, 77 K, and RT).

To obtain continuous temperature variation with the apparatus in Figure 7a, researchers adopted natural evaporation method of LHe inside cryostat before indentation [48]. With the LHe level went down below specimen, the temperature of specimen rose gradually due to the heat condition from the outside. Figure 7b shows the temperature rise of the specimen with time. It can be noticed that there was a temperature rapidly rising stage after LHe evaporating completely, where the maximum rising rate was 2.67 K/min. This is not conducive to conducting repeated experiments at specific temperature, and the indentation process should be carefully controlled to avoid the experimental error induced by temperature variation. Similarly, another temperature rapidly rising stage with larger temperature span also occurred between 100 K and 293 K, which was not mentioned in the research. Thus, the uncontrolled temperature rising rate limited the usability of hardness testing under continuously variable temperature. 

In general, the low temperature indentation method realized with system immersed in cryogenic liquid is advantageous to realization hardness testing at specific temperature. The apparatus is considerably simple and the essential assembly only contains cryogenic liquid, insulated vessel, and a conventional indentation setup. However, the natural boiling point limits the application of the method on variable temperatures. More importantly, the tests conducted by the system immersed in cryogenic liquid were limited to hardness testing, which lacked the measurement of indentation depth, inducing the absence of more mechanical properties obtaining from *P-h* curves.

### 3.2. Contact-Type Low Temperature Testing

**Figure 8:**
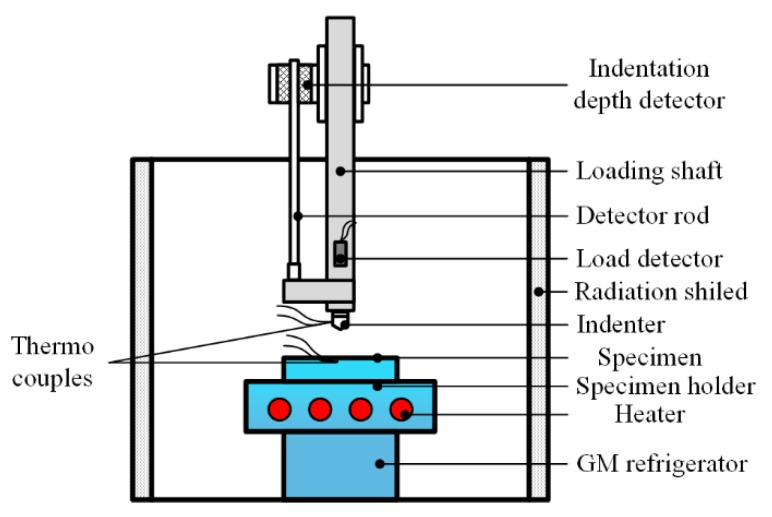
Schematic diagram of indentation apparatus with Gifford-McMahon (GM) refrigerator.

To avoid the shortcomings of directly immersion method, researchers tried to adopt indirect methods to realize indentation at controllable low temperatures. Researchers from Iwate University improved the apparatus in Figure 7a with the ability of conducting indentation at continuously variable temperatures [49], as shown in Figure 8. The same mechanical loading setup was adopted in the apparatus except for an additional differential transformer parallel to the loading shaft, which deformed with the motion and measured the displacement of the indenter. The specimen was fixed on the specimen holder (with heaters inside) which was connected to the 2nd stage of a Gifford-McMahon (GM) refrigerator. A thermocouple was glued on the side of the specimen with Ag paste bond grease to control the testing temperature via GM refrigerator and heaters. The lowest cooling temperature can reach 40 K, and the accuracy of temperature controlling was within ±1 K. Meanwhile, an additional thermocouple was placed onside the indenter, which only played a role in monitoring the temperature variation of indenter. For this reason, a problem of heat flow from indenter similar with apparatus in Figure 6a occurred, influencing the temperature around indentation area. 

The driving mode of the indentation apparatuses introduced above were all based on electric motor (improved from hardness tester), inducing that the indentation load was limited at newton level. With the development of a modern DSI apparatus during recent two decades, the resolution of displacement and load of low temperature indentation were increased accordingly. 

**Figure 9:**
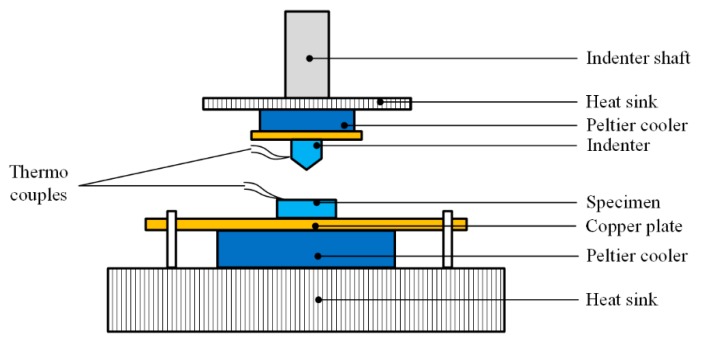
Schematic diagram of depth-sensing indentation (DSI) apparatus with Peltier cooler.

Pethica et al. combined electrostatic driving method (Figure 3) with Peltier coolers, realizing nanoindentation at low temperatures [50], as shown in Figure 9 (driving setups not shown). The indenter and the specimen were, respectively, fixed on two separated Peltier coolers through copper plates. As the working of the Peltier coolers were based on thermoelectric effect, heat sinks were placed behind the Peltier coolers to cool down the high temperature side. The temperature of the specimen and indenter can be simply controlled via adjusting the supplied DC voltage of the Peltier coolers. The mass of the Peltier set besides the indenter was lightweight enough to avoiding the effect during the indentation process.

However, the apparatus in Figure 9 was open, and ice occurred during the cooling process when the temperature fell below 0 °C. Chenc et al. improved the apparatus [51] with: (1) setting the whole apparatus into a sealed chamber purging with argon gas inside to prevent water vapor condensation; and (2) substituting the heat sink with water cooler to achieve better cooling effect. The advantage of adopting Peltier coolers was convenient and low cost, while the temperature of the indenter and specimen could be controlled independently to be identical. However, the cooling power of miniature Peltier coolers limited the testing temperature above 220 K, which was the highest among the several methods. 

**Figure 10:**
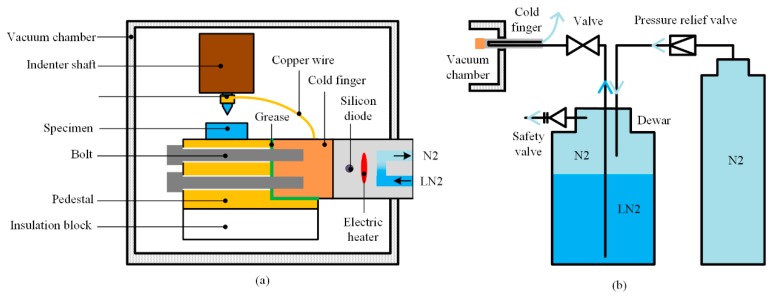
Schematic diagram of DSI apparatus with cold finger (**a**) indentation setups inside vacuum chamber (**b**) cooling system [33].

To obtain a larger temperature range at the condition of continuous temperature variation, Zhao et al. adopted commercial cold finger to realize the application of a low temperature environment down to 150 K [33], as shown in Figure 10a. The whole mechanical setups were set in a vacuum chamber to prevent the water vapor condensation. The occurrence of ice could be effectively prevented at vacuum degree below 10^−2^ Pa. Lee et al. adopted a scanning electron microscope (SEM), providing a vacuum environment and realized in-situ indentation observation ulteriorly [52]. The working principle of the cold finger is shown in Figure 10b. Nitrogen with controlled pressure stimulated LN_2_ in dewar to flow into a vacuum jacketed transfer line, which insulated heat exchange of LN_2_ and carried LN_2_ into the flow path of the cold finger. The flow controllable LN_2_ absorbed the heat of cold finger and was excreted outside the vacuum chamber in nitrogen state. 

An electric heater and silicon diode were set inside the end of the cold finger to realize PID control of the temperature within ±0.05 K. However, the setup of cold finger was too cumbersome to be contacted with the indenter directly. Researchers bonded the end of the cold finger and indenter with a section of copper wire to cool down the indenter to a certain extent. For this reason, the temperature of indenter could not be adjusted finely, and the difference between indenter and specimen was inevitable. Other means should be taken to avoid the related effect, which will be introduced in the next section. 

## 4. Influence Factors and Management

The development of nanoindentation technology was based on the high accuracy of data acquisition, especially the indentation *P-h* curves. For this purpose, certain kinds of works, including calibration of sensors, correction of flexibility of apparatus, and area function of indenter, should be conducted before the indentation process [53,54,55]. Meanwhile, a series of calculation was conducted based on *P-h* curves to obtain mechanical properties, as introduced in Section 2. However, the additional low temperature environment usually affected the accuracy of acquisition and processing of nanoindentation data. The influence factors mainly reflected on three aspects: thermal drift, variation from indenter, and variation from specimen.

### 4.1. Thermal Drift

In nanoindentation testing, the measurement of true indentation displacement *h* is always difficult when comparing with load data. Under normal working circumstances, the determination of displacement only relies on indirect measurement of the displacement of one certain component in the apparatus (center plate and baffle in Figure 3 and Figure 4, respectively) but not the direct movement of the tip of indenter. Thus, the transference of the displacement from indenter tip to the certain component can be easily affected by an external environment, especially temperature influence, which is defined as thermal drift. Thermal drift is induced from thermal expansion occurring somewhere within the apparatus and accumulated with time. Generally, thermal drift can be split into two separate contributions: frame drift and contact drift. 

Frame drift occurs due to temperature variations in the nanoindentation system frame away from the indentation contact area [56]. At room temperature, the frame drift can be effectively reduced through adopting a sealed cabinet outside the apparatus. At low temperatures, frame drift occurs due to thermal gradient of the active and passive cooling components and gradually decreasing temperature of sensors. The former generally decreases over time until stable thermal gradient of each components is established. The latter requires effective isolation between cooling components and sensors, which works within operating temperature range. In both cases, frame drift decreases over time until elimination. Nanoindentation tests should be conducted primarily at the condition of zero-drift status. However, as frame drift is independent of indentation area and information of materials, short period nanoindentation tests are considerable if the frame drift rate performs linear as a function of time.

**Figure 11:**
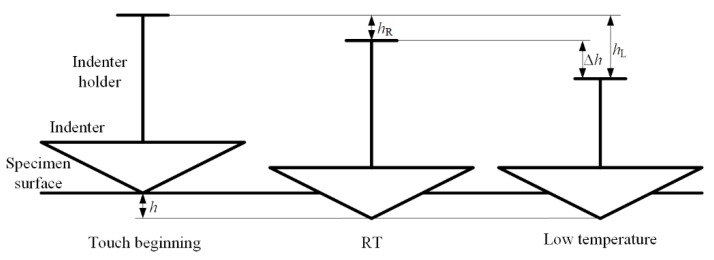
Occurrence of contact drift during low temperature nanoindentation. RT—room temperature.

**Figure 12:**
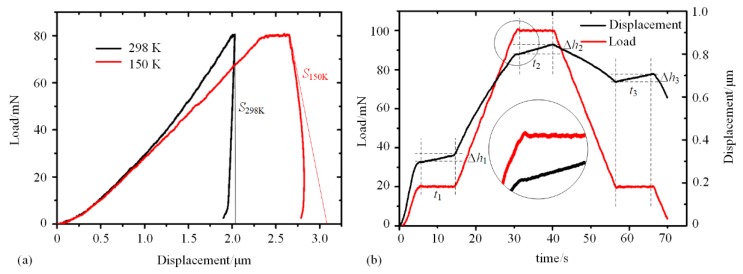
Indentation curves with contact drift. (**a**) *P-h* curve at RT and low temperature. (**b**) h-t curve and *P-t* curve with contact drift.

Contact drift occurs due to thermal deformation of the indenter during the nanoindentation process. Heat flow occurs between indenter and specimen at the contact area, induced by different temperatures between them [57,58]. At RT, the temperatures of indenter and specimen are same; thus, contact drift is negligible. In low temperature nanoindentation, if the indenter is not cooled or the cooling effect is inadequate, the occurrence of contact drift will perform during contact, as shown in Figure 11. With the contact, heat flows from the indenter with higher temperature to the specimen, inducing the length of indenter decreased with temperature reducing. In order to keep the load constant in the moment, the indenter has to be driven downward to keep the contact area. Thus, the nanoindentation *P-h* curves obtained with contact drift in load control mode will performed as shown in Figure 12a. 

Figure 12a shows indentation curves on copper at 150 K with and without contact drift, respectively. The distance between the two curves under a certain load expresses the value of contact drift, which is accumulated over time. Every point in the *P-h* curve with contact drift becomes unreliable, and even negative stiffness occurs during the unloading process, which is impossible for copper material. To measure the rate of contact drift, researchers generally conduct periods of holding time during the indentation process, as shown in Figure 12b, with calculation formula:

(10) $$D=\frac{\Delta h}{t}$$

where *D* is thermal drift rate, and Δ*h* is increment of indentation depth detected by displacement sensor during holding time t. The value of *D* in low temperature nanoindentation is generally positive, while it is negative in high temperature nanoindentation.

The amount of heat flow through the contact depends on the real time contact area, and initial temperature difference between indenter and specimen, which are impossible to be determined through experimental method. It is also inappropriate to adopt later correction after indentation to eliminate the contact drift. Additionally, the heat flow also causes temperature variation of the specimen in the contact area, which affect the actual testing temperatures. Thus, researchers adopted several methods to reduce the temperature difference for eliminating the contact drift through physical means. 

In the immersed cooling method (Figure 6 and Figure 7), the indentation region was set inside the same media. The temperatures of the indenter and specimen are absolutely the same, and no contact drift needs to be eliminated. 

In the Peltier cooling method (Figure 9), two separated Peltier coolers were adopted to cool down the indenter and specimen. Even though the cooling effect of Peltier cooler was affected by heat dissipation, which is generally weakened over time, the temperatures of the two components could be well adjusted combining with thermal couples and DC voltage under PID control. Thus, the Peltier cooling method has the dominant advantage in the aspect of contact drift controlling. 

In the cold finger cooling method (Figure 10), the specimen was exclusively cooled. Thus, the contact drift was larger than the other two methods. The cooling copper wire connecting the indenter and cold finger could only reduce the temperature difference to a certain extent, as shown in Table 1 [33]. To eliminate the contact drift, researchers kept the indenter in touched with the specimen for a period of time at a certain load, which is much higher than subsequent indentation load (generally more than 30 min). The touching time was generally extended with decreasing temperature. Combined with copper wire and pre-touched method, the thermal drift could be basically eliminated. However, the operation of the two methods was complex so that the shape of the copper should be carefully adjusted to avoid influence on the indentation process, and the cooling effect of pre-touched was not permanent, inducing multiple times of the pre-touch operation even under one certain testing temperature. 

### 4.2. Temperature Influence on Indenter

Generally, researchers focused on the elimination of thermal drift in temperature nanoindentation to obtain accurate *P*-*h* curves and considered the indenter as constant. However, in well-controlled low temperature nanoindentation testing, both the specimen and indenter were in cold condition. The property variation of indenter also had influence on the experimental results, which should be fully considered. 

#### 4.2.1. Indentation Hardness

**Figure 13:**
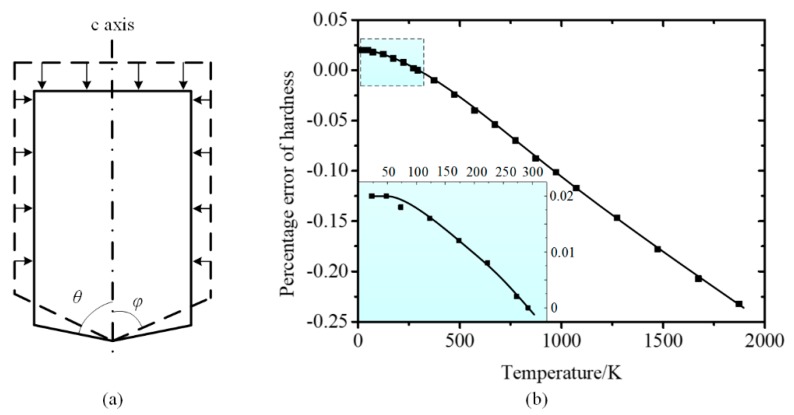
(**a**) Thermal deformation of sapphire indenter at low temperatures. (**b**) Percentage error of hardness at different temperatures [33].

The sapphire indenter had lower heat conductivity compared with diamond indenter and performed better in reducing thermal drift. However, as the sapphire indenter was a single crystalline with hexagonal system, the difference of thermal expansion rate between parallel and perpendicular directions to the c axis could induce shape variation at non-ambient temperatures, as shown in Figure 13a. For an equivalent conical indenter, the thermal expansion rate at perpendicular direction to the c axis is considered uniform. At non-ambient temperatures, the contact radius r deduced from indentation depth can be calculated as:

(11) $$r=\frac{\gamma_{∥}}{\gamma_{⊥}}r_{0}$$

where $\gamma_{∥}$ and $\gamma_{⊥}$ are the linear expansions at parallel and perpendicular directions to the c axis, respectively, and *r*_0_ is contact radius. Under the circumstance that assume the properties of testing material independent with temperature, the error of hardness $\xi_{H}$ can be calculated as follows:

(12) $$\xi_{H}=\frac{\gamma_{∥}^{2}}{\gamma_{⊥}^{2}}-1.$$

The calculated percentage error of hardness induced by thermal expansion anisotropy is presented in Figure 13b [33]. At low temperatures, the maximum error is ~ 0.02%, which is basically negligible to affect the hardness test. However, the calculated hardness is lower than the true value with temperature continued increasing, due to the decrease of equivalent half cone angle. Thus, the area function of the indenter at non-ambient temperatures requires careful consideration to calibrate the hardness calculation.

The occurrence of nanoindentation hardness testing error proceeds from inaccurate estimation of area function of indenter, inducing the *P*-*h* curves deviate from the accurate value. However, this error only occurs in DSI testing. In the situation of micro-hardness testing, the contact area is directly measured after the whole indentation process, which is less depending on the shape of the indenter. However, in most hardness testing the determination process of residual impression area is conducted at RT. The temperature rising process will inevitably lead to thermal deformation of specimen and residual impression, while the coefficient of thermal expansion is various due to different material. Thus, from this point of view, the DSI method is more suitable for hardness testing.

#### 4.2.2. Elastic Modulus

Through formula 9 in Section 2.2.2, it can be seen that the calculation of elastic modulus of specimen is based on the known properties of testing indenter, $\nu_{i}$ and $E_{i}$. The elastic modulus E of material calculated from indentation experiments can be performed as follows:

(13) $$E=\frac{1-\nu^{2}}{\frac{1}{E_{r}}+\frac{1-\nu_{i}^{2}}{E_{i}}}$$

In the case of non-ambient temperature situation, the error of calculated elastic modulus $\xi_{E}$ can be performed as follows:

(14) $$\xi_{E}=\frac{\frac{1}{E_{r}}-\frac{1-\nu_{i0}^{2}}{E_{i0}}}{\frac{1}{E_{r}}-\frac{1-\nu_{i}^{2}}{E_{i}}}-1$$

where *ν*_i0_ and *E*_i0_ are Poisson’s ratio and elastic modulus of indenter at RT, respectively. The value of *ν*_i0_, *ν*_i_, and *E*_i_ are considered as constants. 

**Figure 14:**
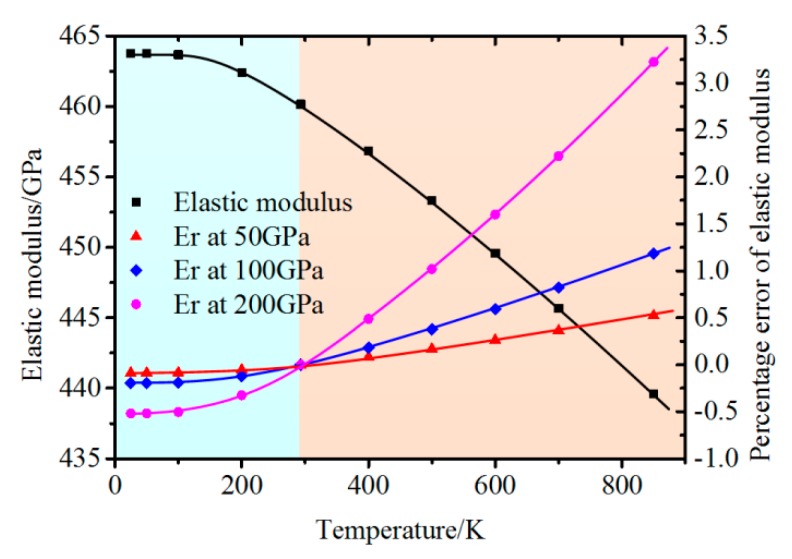
Percentage error of calculation elastic modulus at different temperatures [33].

The calculated percentage error of elastic modulus induced by modulus variation of sapphire indenter is presented in Figure 14 [33,59]. The error increases from negative to positive value with temperature increasing from low to high temperature. More interestingly, $\xi_{E}$ performs much higher in specimen with high elastic modulus. At the condition of 200 GPa, the percentage error value can reach 3.22% at 850 K and −0.52% at 50 K, respectively, illustrating that considerable error occurs in elastic modulus calculation of hard brittle materials. Thus, the influence of elastic modulus variation of tip at non-ambient temperatures should be carefully considered. 

### 4.3. Influence from Specimen

**Figure 15:**
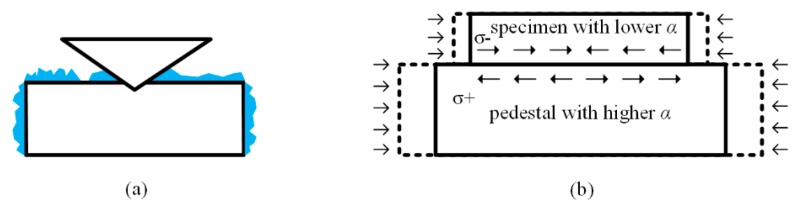
Possible influences occurred on specimen (**a**) ice on the surface (**b**) internal stress.

The most common influence on the specimen is the occurrence of ice under circumstances of a water vapor containing environment, which is an apparent phenomenon on the surface of specimen, as shown in Figure 15a. The thickness of the ice occurred on the free surfaces of the specimen increases with temperature decreasing and time passing by, and it can reach millimeter level in a conventional environment. The existence of ice, even at nanometer level, is unacceptable in nanoindentation testing, due to the confusion of obtained properties of specimen and ice. Researchers usually adopt a vacuum chamber or sealed chamber purging with dry gas to prevent the occurrence of ice. An environment of 10^−2^ Pa can effectively reduce the water vapor content and eliminate the ice occurrence. However, it should be noted that the pressure inside the sealed chamber should be positive to obtain an acceptable effect. The gas under normal pressure could not be dried sufficiently to prevent condensation, which is quite similar with oxidation in elevated temperature nanoindentation. 

Internal stress of specimen is another potential influence factor on testing accuracy during low temperature nanoindentation. With temperature decreasing, both the specimen and pedestal perform cold contraction, and the difference of thermal expansion coefficients could induce intense internal stress inside the specimen, as shown in Figure 15b. For instance, copper is usually selected as the basic material of pedestal, with a thermal expansion coefficient of 16.7 × 10^−6^ K^−1^ and 10.5 × 10^−6^ K^−1^ at RT and 100 K, respectively, while the value of silicon (as a specimen) is 2.5 and 0.5 × 10^−6^ K^−1^ at the same temperatures [59]. The difference of thermal expansion of the two connected units could be a multiple of ten and induces million Pascal level internal stress of specimen. Cryogenic glue between the specimen and pedestal could reduce stress transference to a certain extent. However, re-fasten is more appropriate after refrigeration under the circumstance of mechanical fixation to release the internal stress. 

The internal stress is caused by thermal expansion in horizontal direction, while the expansion in vertical direction could induce position changing of the specimen surface. The thickness of the specimen and pedestal varies with temperature fluctuating. For a copper pedestal, the changing rate induced by vertical thermal expansion could reach over 10 nm/mm·K (10 nm of the vertical position of specimen surface per 1 mm thickness pedestal and 1 K) at low temperatures, which totally reflects to the measurement of indentation depth. Actually, this expansion is one kind of frame drift and could accumulate but not be stable automatically over time. The most direct method to inhibit the drift is enhancing the temperature controlling to reduce temperature fluctuation. Meanwhile, setting the fixed place of the pedestal on the same plane of the pedestal surface could not prevent the thermal expansion but is helpful to reduce the position variation of surface on vertical direction. 

## 5. Applications

### 5.1. Metallic Materials 

**Figure 16:**
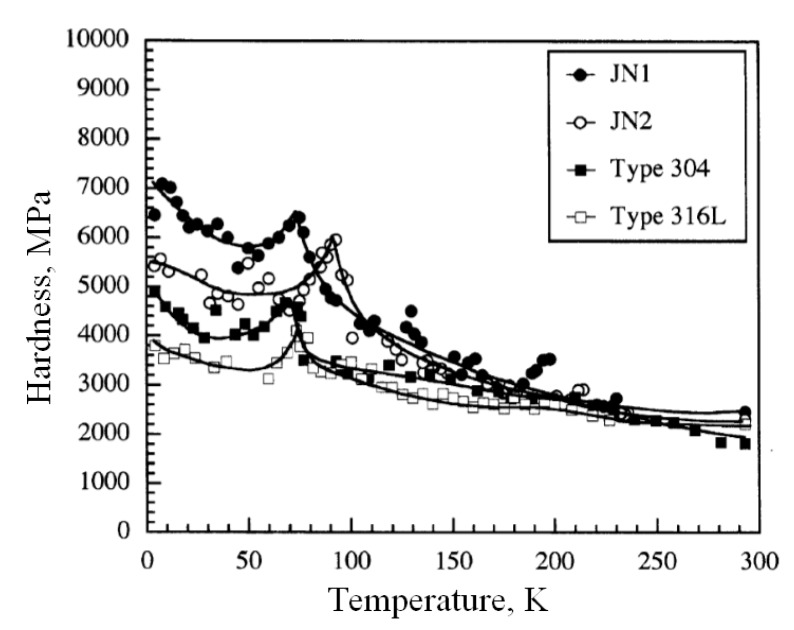
Temperature dependence of hardness for austenitic stainless steels [63].

The most fundamental functionality of low temperature indentation apparatuses could be hardness testing at varying low temperatures [60,61,62]. Yoshino et al. conducted indentations on a series of metallic materials at temperatures from RT to LHe temperature [63]. Their data of austenitic stainless steels are shown in Figure 16, where the observed hardness is plotted as a function of temperature. The data clearly shows that all of the hardness of austenitic stainless steels first increases and then decreases with temperature decreasing, and then further increases below ~ 50 K. A peak value occurs in the range between 50 K and 100 K. This is due to the inhibition of extension of dislocations beneath the indentation region by low temperatures, and the plastic deformation mode transforms from dislocation to twinning. Meanwhile, a phenomenon of ridges occurs inside the residual impressions at low temperatures, which is considered as the external expression of twinning occurrence. The ridges also perform most clearly at temperature range between 45 and 150 K, and turns indistinct both with temperature increasing and decreasing. 

**Figure 17:**
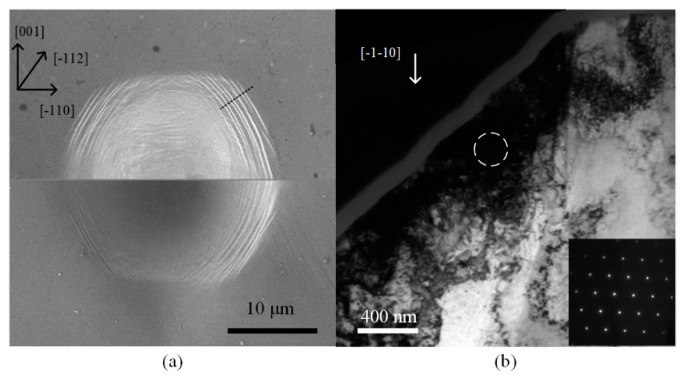
Morphology of (110) copper indentation at 150 K. (**a**) SEM result. (**b**) TEM result at the (**a**) cutting direction [64].

However, the structure of the ridges of austenitic stainless steel beneath the indentation surface was not determined directly. This work was demonstrated by Wang et al., who conducted indentation experiments on monocrystalline copper with spherical indenters at temperatures ranging from RT to 150 K [64]. Ridges only occurred distinctly at 150 K and distributed strictly along the intersecting lines of slip plane and specimen surface, as shown in Figure 17a. The subsurface region of the ridges is shown in Figure 17b, indicating that only monocrystalline structure occurred at 150 K. The ridges were considered as inner slip bands simply caused by increased hardness of material with temperature decreasing and a characteristic of accumulation of geometrically necessary dislocations (GNDs). The inner slip bands resisted the flattening under high pressure and remained after the unloading process. 

**Figure 18:**
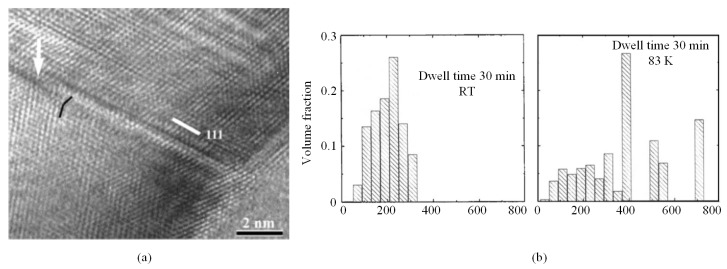
Low temperature indentations on copper [65]. (**a**) Twinning was observed at 77 K. (**b**) Grain size under indentation at RT and 83 K [66].

With temperature decreasing to 77 K, twinning was observed in copper by Gai et al. [65], as shown in Figure 18a, and the dislocations were fewer than RT. More phenomena were found with condition of long time dwelling. The data shown in Figure 18b [66] exhibit grain distribution under indentation region of nanocrystalline copper with dwelling time at RT and 83 K, respectively. Grain growth was more striking at LN_2_ temperature, indicating that the growth of grains is primarily stress, but it is not diffusion driven. The specific reason of this phenomenon is not yet clear. Meanwhile, dwelling also caused hardness decrease with temperature decreasing in various grain size copper [67], which was more likely caused by less creep deformation during dwelling time. In general, most of the property variation and different phenomenon at low temperature of copper was due to the change of plastic deformation mechanism: inhibition of dislocation extension. 

**Figure 19:**
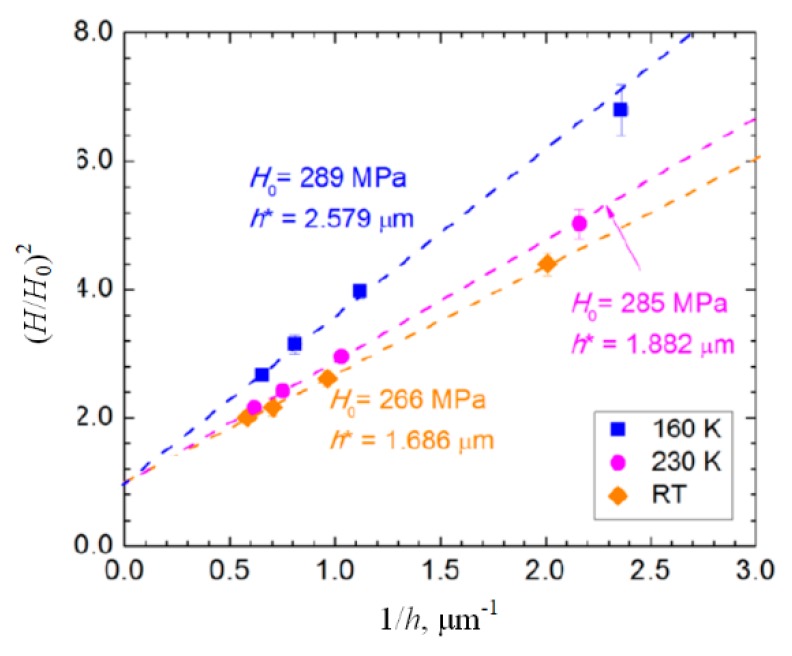
Indentation size effect of (001) Al at RT and low temperatures [68].

More monocrystalline materials with face-centered cubic (fcc) and body-centered cubic (bcc) structure were indented by Lee et al., who conducted nanoindentations and obtained indentation size effect (ISE) from RT to 160 K [68]. The data of (001) Al are shown in Figure 19, where the trend of the ISE was effected by low temperatures. In previous elevated temperatures, Franke found similar phenomenon on copper and argued that the higher dislocation mobility at higher temperatures leads to large GND storage volume, inducing different ISE response at various temperatures [69]. However, Lee et al. considered a highly significant dynamic recovery of dislocation annihilation in fcc materials at relative high temperatures. Meanwhile, only bcc materials were influenced by temperature variation on infinite indentation depth, *H*_0_, which was explained by the amplification in the intrinsic lattice resistance with temperature reduction. 

### 5.2. Semiconductor Materials

**Figure 20:**
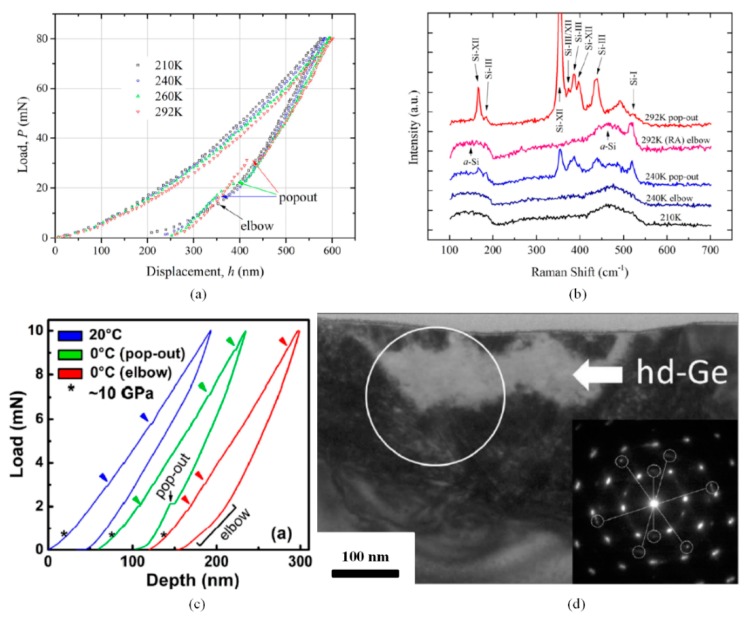
Low temperature indentation on monocrystalline semiconductors. (**a**,**b**) *P*-*h* curves and typical Raman spectra of indented silicon [74]. (**c**,**d**) *P*-*h* curves and TEM result of indented germanium with pop-out [75].

Semiconductor materials, especially represented by silicon, is always research focus in nanoindentation testing field for the various phase state under different high pressures. It is well accepted that the original Si-I phase transforms into a much denser Si-II phase during the indentation loading process, and then transforms into Si-III/XII (slow unloading rate) or a-Si (rapid unloading rate) during the unloading process, with phenomenon of pop-out and elbow, respectively [69,70,71,72,73]. The same as other materials, phase transformation of monocrystalline silicon was both pressure- and temperature-driven. Wang et al. conducted nanoindentations on silicon at temperatures ranging from RT to 210 K, finding the pop-out phenomenon gradually disappeared and substituted by elbow with temperature decreasing [74], as shown in Figure 20a. The phase state of residual impressions conducted at different temperatures was identified via Raman spectra (see Figure 20b), revealing that low temperatures inhibited the transformation from Si-II phase to Si-III/XII, and a-Si could occur even at a slow unloading rate, which strictly leads to pop-out at RT. 

Compared with silicon, germanium is generally considered to have no phase transformation under nanoindentation with a spherical indenter at RT. However, Husto et al. found both pop-out and elbow could occur at 0 °C during the unloading process [75], as shown in Figure 20a, and with temperature further decreasing, which was quite similar with silicon, the probability of elbow occurrence increased and substituted for pop-out phenomenon. Further TEM results (see Figure 20b) revealed that less defect propagation at low temperatures prompted the region under indenter reaching the critical pressure and transformed into r8-Ge or a-Ge during unloading. 

Both the investigations of monocrystalline silicon and germanium above benefitted from the obtained *P*-*h* curves, which performed typical phenomena during the nanoindentation process. Besides pop-out and elbow, other phenomena, including but not limited to pop-in, creep, and serrated flow, provided consecutive information of state variation under indentation region and critical information on the behavior of materials under high pressure. By comparison, before the DSI technology was applied on low temperature indentation, most investigations concentrated on the final state of the residual impressions. Khayyat et al. conducted low temperature indentations on monocrystalline silicon through micro-hardness testing method which consisted of Raman spectra [76,77]. However, due to the absence of mechanical state obtained from *P*-*h* curves, the intermediate Si-II phase was mistakenly considered nonexistent during the indentation loading process. The information obtained from residual impressions was not sufficient to reconstruct the specific load and unload indentation process. 

### 5.3. Ceramic Materials

**Figure 21:**
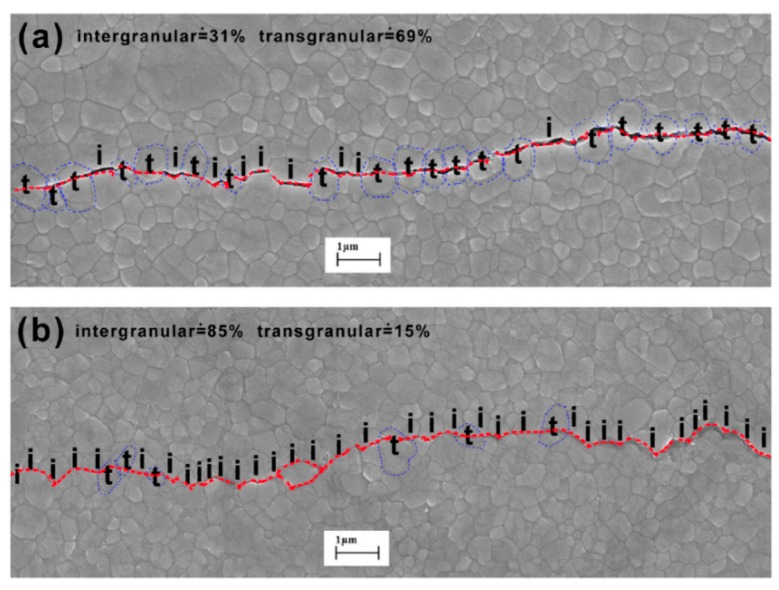
Crack path induced by indentation on the surface of yttria-stabilized zirconia at (**a**) 293 K and (**b**) 77 K [78].

Compared with metal and semiconductor materials, the low temperature indentation research on ceramic materials were focused on the fracture, which were conducted via Vickers indenter with micro-hardness testing method [78,79,80]. The most attractive and practical investigations concerned the crack propagation path generating from indent corners and how it was affected by temperature variation. Figure 21 shows SEM figures of crack path made in yttria-stabilized zirconia at RT and 77 K, respectively [78]. One striking feature is the distinct difference in the fracture mode, transforming from dominantly transgranular fracture to intergranular mode. This indicates that grain boundaries become more brittle at low temperatures, which may be harmful to the fracture toughness. However, the internal mechanism of the mode transformation was not clearly revealed and not extensive enough to draw firm conclusions. This problem also exists in other kinds of ceramic materials, which perform different mechanisms at low temperatures. 

On the other hand, the subsequent analysis on the regions around cracks was essential after indentation, including TEM, Raman spectra, and X-ray diffraction (XRD), which perform information at nanometer, micrometer, and millimeter level, respectively. In the yttria-stabilized zirconia work, researchers used TEM, finding larger transformed martensite with monoclinic structure on either side of the propagating crack at low temperatures. Meanwhile, Raman spectra was utilized to estimate the tetragonal to monoclinic transformation within zones around cracks, and the results of XRD assisted to calibrate the data from Raman spectra. The combination of these subsequent analysis methods provided direct evidence of microstructure evolution during crack propagation and even helped researchers obtain more data for calculation.

## 6. Summary and Perspectives

Low temperature nanoindentation technique has been developed during the past three decades, from micro-hardness to DSI method, and realizes temperature down to 4.2 K (LHe). Several cooling methods have been attached to the conventional indentation apparatus, such as cryogenic liquid immersion, Peltier coolers, directly evaporative cooling, and cold finger. Each cooling method has advantages and disadvantages on variable temperature ability, temperature range, temperature stability, and convenience of test (see Table 2). Indentation influence factors, especially contact thermal drift, have been considered and eliminated during low temperature nanoindentation testing. Based on the according apparatus, researchers conducted low temperature indentations on different kinds of materials, and numerous phenomena and trends were discovered distinct from RT. However, apart from the achievement we mentioned above, there are still a number of perspectives which could be considered in future investigation of low temperature nanoindentation technique.

Firstly, and most importantly, the temperature range and operability of elimination of contact thermal drift need to be improved to realize stable nanoindentation at low temperatures and simplify the preparation before nanoindentation. An improved method that could achieve the two advantages simultaneously is meaningful to low temperature nanoindentation. From this point of view, adopting a low temperature atmosphere environment to cool down the specimen and indenter may become a more suitable method. On the other hand, most of the current experiments conducted nanoindentation at a constant temperature, while temperature change during holding the indenter at a specific load to investigate the effect of temperature variation on materials under complex stress condition has not been reported. However, this operation is extremely difficult to realize as the control of frame thermal drift should be controlled within nanometer level.

Secondly, ultra-low load (μN) nanoindentation, which has been well realized at RT, is anticipated in a low temperature environment. This needs the influence generated from cooling system to be virtually eliminated to allow the load and displacement sensors to obtain data with low enough noise. The existing electrostatic driving method, atomic force microscopy (AFM), and Micro-Electro Mechanical System (MEMS) could conduct as the indentation driving device, while the latter two methods even have the ability to reach nanometer level impression. Besides load aspect, ultra-low temperature (mK) is also desirable. However, the environment of such a low temperature is usually enclosed, which is contradictory to the motion components of indentation apparatus and unfavorable for indentation sensors.

Thirdly, the subsequent tests after low temperature nanoindentation need to be optimized. In the current experimental sequence, the specimen is usually taken out of the indentation environment and subsequent tests are conducted, such as SEM, TEM, XRD, and micro Raman spectra, in an ambient environment, which may cause additional property variation with temperature rising. A corresponding cooling system attached to this testing equipment, as well as transportation between indentation area and subsequent test area, is beneficial to maintain the original state of the specimen after low temperature nanoindentation, as well as enhance the credibility of experimental conclusions.

Lastly, the current target in development of low temperature nanoindentation is realization of the functions which RT test has, including continuous stiffness measurement (CSM), scanning probe microscope (SPM) imaging, property mapping, and even scratch test. This will require a cooling system performing better compatibility on the additional functional modules and further reduce the thermal drift and other influence factors. Compared with standardized and relatively simple operation in RT nanoindentation, a highly skilled and knowledgeable user is still currently required to complete the variable low temperature operation. By implementing the above techniques, it is believed that low temperature nanoindentation technique will be more widespread as an advanced testing method, inheriting the rapid and high-precision properties of conventional nanoindentation technique. This will facilitate the investigation on the material behavior under both low temperature and high stress/strain conditions.

## Figures and Tables

**Table 1:** Thermal drift rate under different cooling methods (nm/s) [33].

Temperature	Original	Copper Wire	Pre-Touched
298 K	0.049 ± 0.005	−0.019 ± 0.008	0.033 ± 0.007
250 K	1.946 ± 0.104	0.383 ± 0.159	0.028 ± 0.005
200 K	3.793 ± 0.344	0.774 ± 0.208	0.063 ± 0.008
150 K	4.985 ± 0.325	1.137 ± 0.222	0.092 ± 0.021

**Table 2:** Advantages and disadvantages of existing cooling methods.

Cooling methods	Advantage	Disadvantages
Cryogenic liquid immersion	• High temperature stability • Natural non-thermal drift	• Immutable temperature • Potential liquid influence
Peltier coolers	• Low cost • Easy to control and quick response	• Limited temperature control range
Directly evaporative cooling	• Large temperature control range	• Uncontrolled temperature rising
Cold finger	• High accuracy temperature control	• Low temperature cooling speed
